# Reliability and Validity of Non-invasive Blood Pressure Measurement System Using Three-Axis Tactile Force Sensor

**DOI:** 10.3390/s19071744

**Published:** 2019-04-11

**Authors:** Sun-Young Yoo, Ji-Eun Ahn, György Cserey, Hae-Young Lee, Jong-Mo Seo

**Affiliations:** 1 Department of Electrical and Computer Engineering, Inter-University Semiconductor Research Center, Institute of Engineering Research, Seoul National University, Seoul 08826, Korea; sweden5656@snu.ac.kr; 2 Interdisciplinary Program in Bioengineering, Seoul National University, Seoul 03080, Korea; dkswldms@snu.ac.kr; 3 Faculty of Information Technology and Bionics, Pázmány Péter Catholic University, 1083 Budapest, Hungary; cserey@digitus.itk.ppke.hu; 4 Department of Internal Medicine, Seoul National University Hospital, Seoul 03080, Korea; hylee612@snu.ac.kr; 5 Biomedical Research Institute, Seoul National University Hospital, Seoul 03080, Korea

**Keywords:** non-invasive blood pressure measurement, radial arterial pressure, continuous blood pressure monitoring, three-axis tactile force sensor

## Abstract

Blood pressure (BP) is a physiological parameter reflecting hemodynamic factors and is crucial in evaluating cardiovascular disease and its prognosis. In the present study, the reliability of a non-invasive and continuous BP measurement using a three-axis tactile force sensor was verified. All the data were collected every 2 min for the short-term experiment, and every 10 min for the long-term experiment. In addition, the effects on the BP measurement of external physical factors such as the tension to the radial artery on applying the device and wrist circumference were evaluated. A high correlation between the measured BP with the proposed system and with the cuff-based non-invasive blood pressure, and reproducibility, were demonstrated. All data satisfied the Association for the Advancement of Medical Instrumentation criteria. The external physical factors did not affect the measurement results. In addition to previous research indicating the high reliability of the arterial pulse waveforms, the present results have demonstrated the reliability of numerical BP values, and this implies that the three-axis force sensor can be used as a patient monitoring device.

## 1. Introduction

Blood pressure (BP) is one of the essential vital signs and abnormal BP usually requires treatment. For example, hypertension is associated with various systemic diseases which can be relieved by the early detection and the control of the high blood pressure. Continuous BP monitoring is indispensable in monitoring patient status during surgery under general anesthesia, in the intensive care unit (ICU) or in the emergency room (ER) [1,2].

Methods of measuring BP can be categorized into invasive and non-invasive modalities. The invasive, arterial line (A-line)/Arterial Blood Pressure (ABP) method uses a catheter inserted directly into the artery and connected to a pressure sensor, and this method is considered the gold standard of continuous BP measurement [3]. However, long-term use of the A-line catheter is associated with local or systemic infection, arterial injuries, and embolism, which might be fatal to the unconscious patient. Due to these complications and pain at the puncture site, medical doctors sometimes hesitate to use A-line in the emergency room or in the alert patient with unstable hemodynamic status.

Non-invasive blood pressure (NIBP) measurement uses a pressurized cuff around the arm or the leg. The auscultatory method [3] measures BP by detecting the sound of the opening and closing of the limb artery. The oscillometric method [4,5,6,7,8,9] catches the vibration of the cuff during pressurized air release. As NIBP can be obtained by sending air pressure to the cuff and releasing it alternately, continuous BP monitoring is impossible. Another NIBP monitoring method, the pulse wave velocity (PWV) measuring device, which is considered to be the gold standard to diagnose the degree of arterial stiffness, needs to be held by pressing the blood vessel with constant force and at the appropriate contact angle.

A non-invasive, continuous and reliable BP measuring device would help monitor patients without pain and the risk of infection in emergency situations such as an accident, in ambulance transfer or in ER. It could also be applied safely and easily to patients undergoing surgery, and moreover, it would be especially beneficial for unconscious patients under continuous monitoring for a long period by reducing opportunistic infection. For this reason, photoplethysmography (PPG)-based, ultrasound-based, and tactile-sensor-based [10] approaches are under investigation. The ultrasound-based method [11,12,13] measures BP according to the difference between the transmitted and reflected ultrasound frequencies passing through an artery. This seems to be straightforward but requires data acquisition and analysis by experts, and moreover, the equipment is bulky and expensive. The PPG-based method [14,15,16] detects changes in the diameter of blood vessels using a near-infrared image sensor without skin contact. However, the measurement results can be affected by the position of the light source, and the capillary pressure monitored by a PPG-based device cannot reflect the full dynamic range of the diastolic and systolic arterial pressure. The tactile-sensor-based method [17,18,19] utilizes applanation principles to measure BP according to the magnitude of the pulsatile force. This method is easy to use and less influenced by other factors such as body temperature, although calibration according to the shape and the position of the sensor is needed.

In this paper, the three-axis tactile force sensor (OMD-20-SE-40N, Onrobot, Denmark) which measures the magnitudes of the *x*-, *y*- and *z*-axis forces, was used. The three-dimensional vector sum of the force monitored by this sensor is reproducible regardless of the position or the contact angle of the sensor to the object, BP can be estimated by placing this sensor onto the skin over the artery with simple calibration. In comparison, applanation tonometric BP measurement is based on the Imbert–Fick law that measures the pressure inside the artery by equilibrating it with the tension of the arterial wall on perpendicular flattening of the convex surface; thus, it is sensitive to the contact angle of the pressure-sensing tip onto the skin over the artery. In addition, if the examinee does not change position during BP monitoring with the three-axis tactile force sensor, then the single largest vector axis can be selected as the main component of interest for calibration and measurement, and this makes BP monitoring much easier. Foldi et al. [18] demonstrated that arterial BP waveforms from the three-axis tactile sensor showed a high similarity with those from a Millar applanation tonometer. They showed the similarity of the waveform between the three-axis tactile force sensor and the sphygmomanometer with qualitative analysis.

In this study, we validated the reliability of the continuous numerical values quantitatively obtained by the three-axis tactile force sensor as an index of the arterial pressure for continuous monitoring. The initial amplitude of the radial arterial pulses measured by the force sensor were matched with the systolic and the diastolic BP measured by the oscillometry-based NIBP device, and the correlation between the amplitude of the pulsatile waves and the intermittent NIBP results were reviewed in the short and long term. Considering the reliability of the tactile force sensor waveforms from Foldi et al. and values from this paper, the NIBP measurement system using a three-axis tactile force sensor may be used as a new state-of-the-art method, and greatly facilitate blood pressure measurement in unconscious patients or in emergencies without being concerned about complications.

## 2. Materials and Methods

### 2.1. Experimental Setting

**Figure 1:**
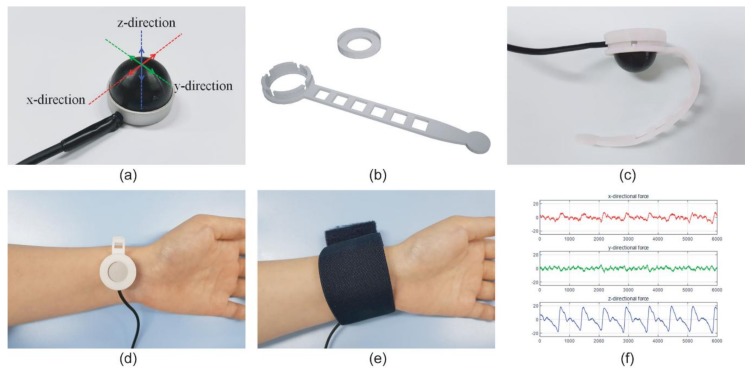
(**a**) The three-axis tactile force sensor; (**b**) Three-dimensional (3D) schematic of semicircular bracelet; (**c**) Semicircular bracelet with three-axis tactile sensor and its application onto the wrist for monitoring non-invasive blood pressure (NIBP) on the radial artery (**d**); (**e**) Elastic wristband over the system to ensure stable position; (**f**) Data from the three-axis tactile sensor with pulse detection.

The dome-shaped three-axis tactile force sensor detects *x*-, *y*- and *z*-axis forces by optical measurement of the dome deformation. Infrared light is reflected on the internal surface of the dome and four photodiodes inside the sensor detect the deviation of the reflected light. This information is transformed into the force vectors of the three cardinal directions [18,20,21]. It is sensitive enough to detect deformation of the dome from hundreds of nanometers up to 3 mm. The sensor dome is made of silicone rubber on a titanium plate, both of which are well-known as biocompatible materials. Because of its shape (Figure 1a), the force sensor should be appropriately held onto the target surface. For this purpose, a polylactic acid (PLA)-based semicircular bracelet (Figure 1b,c) was made using a fused deposition modeling (FDM)-based 3D printer (3DWOX DP200, Sindoh, Seoul, Korea). Since the PLA filament is thermoplastic, it can be easily deformed in hot water even after having been printed in a flat conformation [22]. To ensure stable monitoring of the radial arterial pulse, an elastic wristband was applied over the system (Figure 1e). The pressure change of the radial artery was recorded (Figure 1f) and compared to the oscillometry-based wrist BP (HEM-6121, Omron, Japan) which satisfies ISO 81060-2:2013.

### 2.2. Subjects

In order to evaluate the reliability of BP values from the three-axis tactile force sensor, short- and long-term measurements were implemented after Institutional Review Board (IRB) approval (H-1809-113-974). In the short-term measurement, 30 volunteers without any cardiovascular problems attended (male: 19; female: 11; mean age: 30 years; range 22–59 years). According to the central limit theorem, the sample size of 30 in the short-term experiment was sufficient to form a Poisson distribution and transform to a normalized distribution for the statistical evaluation. After verifying the reliability of the short-term data, 10 of these volunteers also participated in the long-term measurement (male: 9; female: 1; mean age: 31.5 years; range 23–59 years). Because the general semiconductor sensors, including the tactile sensor, could maintain their characteristics continuously—under the assumptions of no environmental temperature or atmospheric pressure changes and no heat stack phenomenon from the USB 2.0 standard microcurrent (5V, 0.1A)—there was no need to retain all 30 volunteers to participate in the long-term experiment. Before conducting the experiments, all volunteers were well-informed about the experiments and agreed to participate.

### 2.3. Procedure

All volunteers met the criteria for the BP difference between each arm being within 10 mmHg on oscillometric NIBP measurement. The three-axis tactile force sensor was positioned over the left radial artery by palpitation and was covered by the wristband, while the oscillometric tonometer was wrapped onto the right wrist. On applying the wristband over the system, the tightness was carefully adjusted to maximize the amplitude of the arterial pulse wave. During the experiments, all volunteers were seated comfortably with both arms on the table at the same level as their heart. Pulse waveforms and values were recorded continuously: for short-term measurement, systolic and diastolic BP from the oscillometric tonometer was measured every 2 min, for a total of 5 measurements; for long-term measurement, the same procedure was repeated every 10 min, for a total of 6 measurements. For both experiments, the initial systolic and diastolic BP from the oscillometric tonometer were obtained and matched with the peak and the trough value of the initial sensor waveform. While the long-term data were being acquired, the examinees were allowed to do some simple activities such as reading; however, they were asked to take the greatest possible care to maintain both hands at heart level.

### 2.4. Signal Acquisition and Post-Processing

**Figure 2:**
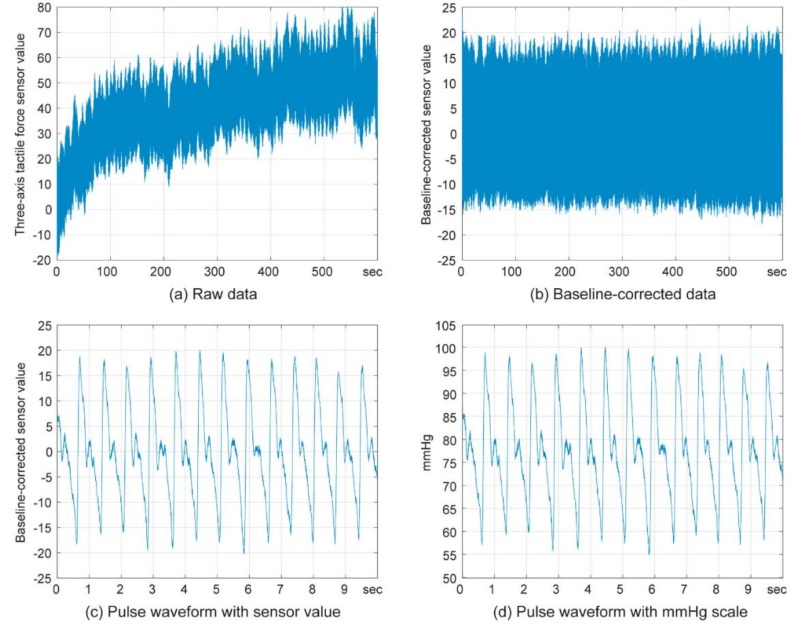
(**a**) Raw data from three-axis tactile force sensor in short-term experiment; and (**b**) the result of baseline correction with the Savitzky–Golay algorithm; (**c**) Recorded pulse waveform with original sensor scale and (**d**) with mmHg scale calibrated by the oscillometric tonometer.

Sensor data was recorded at the sampling rate of 1000 Hz with a 15 Hz filter to remove the 60 Hz AC power noise. All of the post-processing was done with MATLAB software (R2018b, The MathWorks, Inc., Natick, MA, USA). There were sudden changes of baseline from hand movement or muscle contraction artifacts, as well as gradual changes caused by internal noise such as power line interference of the sensor in the raw data. To overcome this instability, baseline correction based on the Savitzky–Golay algorithm was implemented in the time domain (Figure 2) by subtracting the polynomial equation obtained according to neighbor values [23,24].

### 2.5. Statistical Analysis

Statistical analysis was performed using SPSS software (version 23, IBM, Armonk, NY, USA). To match the systolic and diastolic BP values with those of the oscillometric tonometer, the averages of the minimum and maximum values, respectively, from the tactile sensor data obtained during 10 s (approximately 13–15 pulse waveforms, varying according to the heart rate), were taken every time the oscillometric tonometer was used. The raw data were affected by the position of the sensor over the radial artery and by the tightness of the supporting wristband in each volunteer. The purpose of this study was the evaluation of the reproducible, continuous NIBP monitoring in the examinees, and the paired analysis between the baseline-corrected sensor data and the reference value was conducted first. After verifying the high correlation between them, linear regression was performed for both the short-term and long-term experiments to evaluate the correlation between the baseline-corrected 3D force sensor values and the cuff-based NIBP, and to obtain a linear equation for estimating BP from the baseline-corrected sensor value.

Even though the tightness of the wristband was carefully adjusted to maximize the amplitude of the arterial pulse wave (measured as the raw numerical value of the sensor), it inevitably varied from person to person, and the correlation between the amplitude of the arterial pulse wave and the NIBP might have been affected by the tightness of the wristband. Thus the difference between the calibrated BP from the sensor and the NIBP was evaluated against the raw numerical value of the sensor (which may reflect the tightness of the wristband) with correlation analysis, and against the wrist circumference in the same way.

To analyze the reproducibility, reliability, and degree of agreement between the two methods, a Bland–Altman analysis and an intraclass correlation coefficient (ICC) analysis were conducted [25,26,27,28,29,30,31,32].

## 3. Results

### 3.1. Linear Regression Analysis

**Figure 3:**
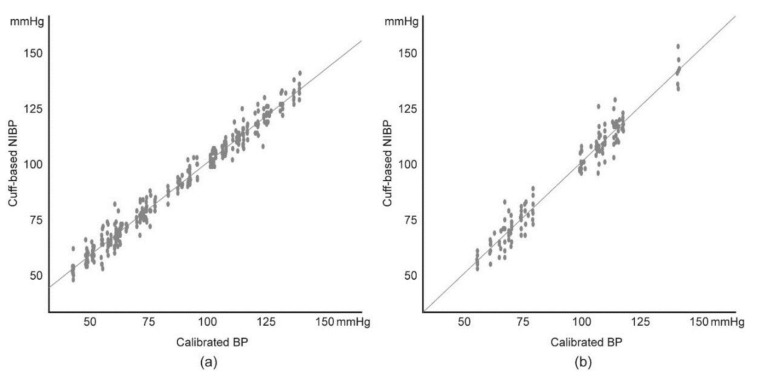
Linear regression between the cuff-based NIBP and the calibrated blood pressure (BP) in: (**a**) short-term experiment and (**b**) long-term experiment. Interpersonal comparison.

In the short-term experiment, linear regression analysis showed high linearity between the cuff-based NIBP and the baseline-corrected 3D force sensor value in each examinee, with an average R^2^ value of 0.970 (Figure 3a, Table 1). The analysis of variance showed good significance (F-test, *p* < 0.05), and the average standardized beta coefficient proved that the 3D force sensor data and cuff-based NIBP had a positive and strong linear relation. Based on these results, all of the raw data from the 3D force sensors were calibrated using the linear regression equation. Then, the calibrated sensor value (calibrated BP) was compared to the cuff-based NIBP in all examinees, and these results also showed a strong linearity (R^2^ = 0.977).

The correlation analysis showed poor linear correlation between the raw numerical value of the sensor and the difference in the calibrated BP and the cuff-based NIBP, in both systolic and diastolic BP. The wrist circumference also showed poor correlation with the BP differences (Table 2). This implies that the tightness of the wristband or the circumference of the wrist does not affect the measurement result of the sensor.

In the long-term experiment, linear regression between the cuff-based NIBP and the baseline-corrected 3D force sensor value in each examinee showed high linearity as in the short-term experiment, and the calibrated BP showed a strong linearity with the cuff-based NIBP in all examinees (Figure 3b, Table 1). The correlation analyses between the raw numerical value of the sensor and the difference in the calibrated BP and the cuff-based NIBP, and between the wrist circumference and those BP differences, also showed the same result as the short-term experiment (Table 2).

### 3.2. Bland-Altman Analysis

**Figure 4:**
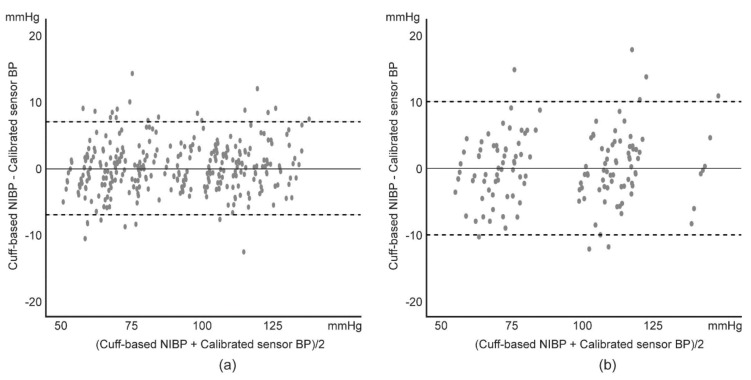
Bland–Altman plot of the calibrated BP and the cuff-based NIBP in: (**a**) short-term experiment and (**b**) long-term experiment.

A one sample *t*-test between the calibrated BP and the cuff-based BP did not reveal a statistically significant difference (*p* > 0.05) in the short-term or long-term experiments (Table 3). Based on the high linearity between the calibrated BP and the cuff-based BP, a Bland–Altman analysis [27,28] was performed to examine discrepancies between the two methods. On the Bland–Altman plot, the bias and variations were distributed almost uniformly over the entire BP range within the 95% limit of agreement (Figure 4, Table 4). Linear regression between the difference of the calibrated BP and the cuff-based BP and the average of those in short-term and long-term experiments did not show a strong linear relationship (Table 5).

### 3.3. Intraclass Correlation Coefficient

The intraclass correlation coefficient (ICC) is an index of repeatability and reproducibility that compares two different measurements directly [30,31,32]. The ICC of the calibrated BP and the cuff-based NIBP showed strong consistency in the short-term and long-term experiments (Table 6).

## 4. Discussion and Conclusions

Continuous BP measurement in a non-invasive way can greatly improve patient monitoring, especially in unconscious or in unstable cardiovascular disease patients. The previous study showed the reliability of the three-axis tactile force sensor as a non-invasive, arterial pulse waveform monitoring system. In the present study, BP values from the three-axis tactile force sensor were proved to be reliable and reproducible in comparison with the oscillometric tonometer. There was a strong linear relationship between the cuff-based NIBP and the 3D force sensor value in both short-term and long-term measurement in a sitting position. This high correlation with the cuff-based NIBP was evident not only as the baseline-corrected raw data but also as the calibrated BP. All the bias and standard deviation (SD) results for systolic, diastolic and mean arterial BP have satisfied the Association for the Advancement of Medical Instrumentation (AAMI) criteria, which state that the bias and SD should be below 5 mmHg and 8 mmHg, respectively (Table 7). Also, the tightness of the arm band and the wrist circumference did not affect the calibrated BP.

A-line measures BP parallel to the direction of the blood flow by inserting a catheter, thus it is the most accurate method although it is invasive. The cuff-based NIBP method measures BP by blocking the blood vessel completely and releasing it, thus the measurement can be easily dropped by the motion artifact, and this takes another 10 or more seconds for the repeat measurement. On the other hand, BP measurement using the tactile force sensor estimates BP with blood vessels in a semi-blocked state. This status does not follow the ideal Imbert–Fick law, since the simple press on the radial artery with the tactile sensor cannot ensure the perpendicular force equilibrium with the tangential arterial wall tension. Additionally, the semi-blocked state may result in lower systolic and higher diastolic BP. However, as the dome-shaped 3D sensor can detect three-axis force, the contact angle with the radial artery does not affect the results significantly. In other words, as the position of the 3D tactile sensor does not affect the calibrated BP, once the sensor detects the pulse, the proposed system can measure the arterial BP waveform and values correctly. Furthermore, we demonstrated the reliability of the calibrated BP values from the 3D force sensor compared with the cuff-based NIBP. These results imply that the proposed method has great potential for clinical application.

## Figures and Tables

**Table 1:** Linear regression between the cuff-based NIBP and the baseline-corrected sensor values.

		R^2^	*p* Value (F-test)	Standardized Coefficient
***Short-term experiment***				
**Intrapersonal results**	**Mean value and mean BP**	0.970	0	0.985
	**Peak value and systolic BP**	0.995	0	0.997
	**Trough value and diastolic BP**	0.913	0	0.956
**Interpersonal results**		0.977	0	0.988
***Long-term experiment***				
**Intrapersonal results**	**Mean value and mean BP**	0.945	0	0.972
	**Peak value and systolic BP**	0.982	0	0.991
	**Trough value and diastolic BP**	0.851	0	0.923
**Interpersonal results**		0.978	0	0.963

**Table 2:** Correlations of degree of pressure on radial artery and wrist circumference with difference between calibrated BP and the cuff-based NIBP.

		Differences in Systolic BP	Difference in Diastolic BP
***Short-term experiment***			
**The raw numerical value of the sensor**	**Pearson correlation coefficient**	0.051	0.023
	***p* value, 2-tailed**	0.790	0.904
**Wrist circumference**	**Pearson correlation coefficient**	0.063	0.010
	***p* value, 2-tailed**	0.741	0.958
***Long-term experiment***			
**The raw numerical value of the sensor**	**Pearson correlation coefficient**	0.248	0.349
	***p* value, 2-tailed**	0.489	0.323
**Wrist circumference**	**Pearson correlation coefficient**	0.122	0.234
	***p* value, 2-tailed**	0.737	0.515

**Table 3:** Results of one sample *t*-test in short-term and long-term experiment.

	*t* Value	Degree of Freedom ^+^	*p* Value, 2-Tailed	Mean Difference	95% Confidence Interval of Difference	
					Lower	Upper
***Short-term experiment***	−0.074	359	0.941	−0.014	−0.382	0.354
***Long-term experiment***	−0.083	139	0.934	−0.036	−0.886	0.814

+ the number of data points estimated (= the number of measurements × 2 (systolic and diastolic) × the number of volunteers) − 1.

**Table 4:** Statistical values of bias between the calibrated BP and the cuff-based NIBP in the short-term and long-term experiments.

	Mean Value (Bias)	Standard Deviation of Bias	Standard Error of Bias
***Short-term experiment***	−0.014	3.548	0.187
***Long-term experiment***	0.036	5.088	0.430

**Table 5:** Linear regression between the difference of the calibrated BP and the cuff-based BP and the average of these in the short-term and long-term experiments.

	R^2^	*p* Value (F-test)	Standardized Coefficient
***Short-term experiment***	0.077	0.143	0.077
***Long-term experiment***	0.109	0.200	0.109

**Table 6:** Results of intraclass correlations on differences in the short-term experiment.

	Intraclass Correlation (95% Confidence Interval)	F-test		
		F Value	Degree of Freedom	*p* Value
***Short-term experiment***				
**Single Measures**	0.988 (0.986~0.991)	170.62	359	0.000
**Average Measures**	0.994 (0.993~0.995)	170.62	359	0.000
***Long-term experiment***				
**Single Measures**	0.977 (0.969~0.984)	87.664	139	0.000
**Average Measures**	0.989 (0.984~0.992)	87.664	139	0.000

**Table 7:** Bias and standard deviation (SD) of systolic, diastolic and mean arterial blood pressure.

	Systolic BP		Diastolic BP		Mean arterial BP	
	Bias	Standard Deviation	Bias	Standard Deviation	Bias	Standard Deviation
***Short-term experiment***	−0.010	5.419	0.062	4.772	0.036	4.610
***Long-term experiment***	−0.021	3.338	−0.006	3.710	0.014	2.924
